# Supplementary material for: Wearable Spectroradiometer for Dosimetry
Source: Sensors (Basel). 2022 Nov 15;22(22):8829. doi: 10.3390/s22228829 (PMC9697616; doi:10.3390/s22228829)
Supplement: Supplementary file 1 [file sensors-22-08829-s001.zip › sensors-1967207-supplementary.pdf]

Back Matter: Wearable Spectroradiometer for Dosimetry

Platform Circuit

Figure S1 displays the electrical connections between the Arduino Due, peripheral devices, and the spectroradiometer, RTC, and microSD interface. Table S1 details all labelled pins in this diagram.

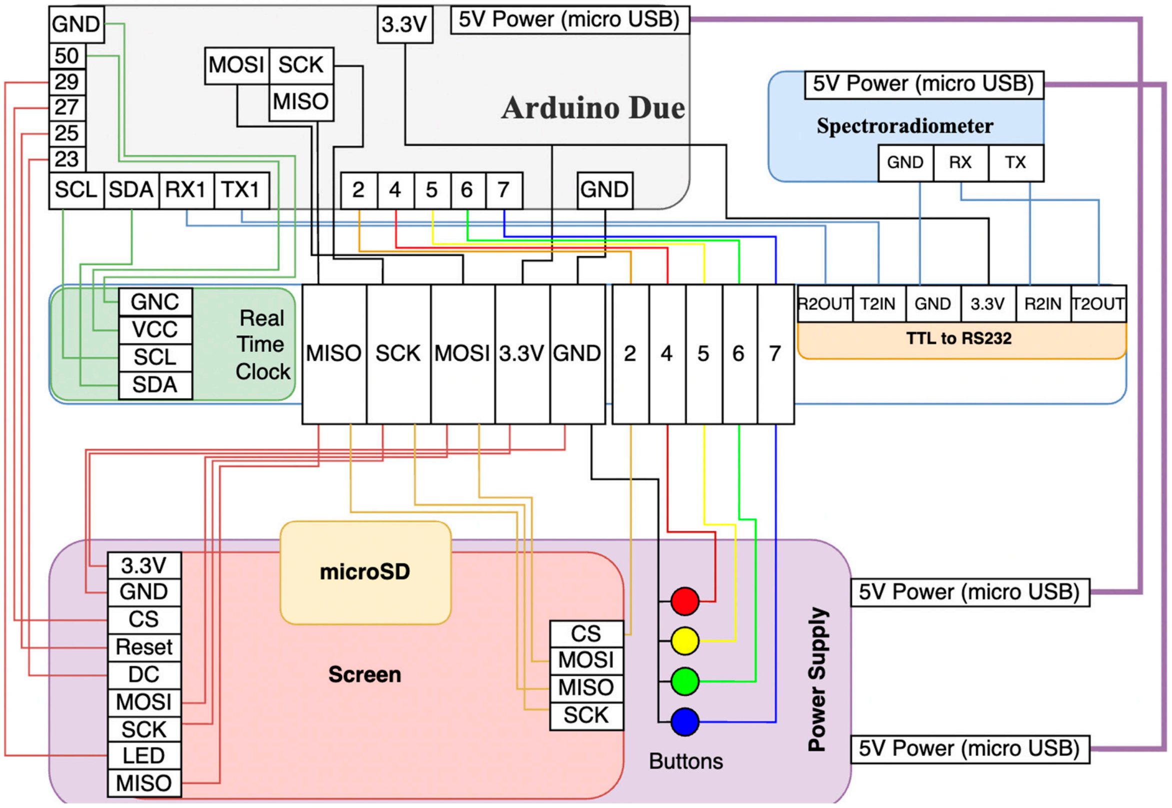

Figure S 1: The wiring schematic for the platform.

Table S 1: Descriptions for each pin label.

Label	Name	Description
5V Power (micro USB)	Power supply Connection	Connect the spectroradiometer and the Arduino Due to the power supply through micro USB ports
3.3V	Voltage High (3.3V)	Connects the peripheral to the 3.3V wire.
GND	Voltage Low (0V)	Connects the peripheral to the platform electrical ground.
MOSI	Master Out Slave In	Connection for the master device to send data to the slave device
MISO	Master In Slave Out	Connection for the slave device to send data back to the master device
SCK	Serial Clock	Line that carries the clock pulse generated by the master device
SDCS OR LCDCS	Chip Select (for SD card or LCD display)	Connection used by the master device to inform the slave device that it will send or request data. The SS/CS pin should be set to LOW to inform the slave that the master will send or request data. Otherwise, it is always HIGH
D/C	Data/Command pin	Switch on the TFT to send data to the SD card or the LCD display
TE	Tearing Effect	optional output from the display to synchronize data writes, avoiding the ‘Tearing Effect' that is seen when data is changed halfway through a display refresh
PWM	Pulse width modulation	Allows the input of a PWM signal to dim the backlight of the LCD display
RX	RS232 Data Receiving Pin	The Recommended Standard 232 pin that sends data from the device.
TX	RS232 Data Transmitting Pin	The Recommended Standard 232 pin that receives data from the device.
Pin #s	Arduino Pins	Multi-purpose pins that allow transmitting or receiving bits to and from the Arduino Due.
SDA	Serial Data Line	Data gets sent through this pin and gets synchronized with the clock signal on the SCL
SCL	Serial Clock Line	Receives the clock signal.

Peripheral Communication

Terminology

The platform primarily uses 16-bit data packets called ‘data words’ to communicate between the microcontroller and peripherals. This data structure can be described as two bytes, with 8-bits representing the large value called the most significant byte (MSB) and 8-bits representing the small value termed the least significant byte (LSB). In infrequent instances, this platform uses 32-bit data packets called double data words, which themselves can be described as two data words. As before, the term for the 16-bits representing the large value is the most significant data word (MSW) and the term for the 16-bits representing the small value is the least significant word (LSW).

The rate at which information is transferred in a communication channel is called the baud rate. A baud rate of 115200 refers to a rate of 115200 bits per second.

RS-232 Standard

This platform uses the Recommended Standard 232 for 115200 baud rate communication between the Arduino Due and the spectroradiometer. The standard formally defines signals connecting between a data terminal equipment, in this case the Arduino Due, and a data connection equipment (the spectrometer). The standard represents bits as positive and negative voltages within a range of +3V to +15V and -3V to -15V [1]. Researchers and engineers commonly opt to use the RS232 serial communication standard to control scientific equipment because of its simple implementation and adaptability.

Spectroradiometer Commands

The spectroradiometer firmware requires commands send via RS232 to begin with an ASCII character sometimes followed by binary data specifying an option. The spectroradiometer returns an electrical acknowledgement (ACK) upon receipt of a successful command and an electrical negative acknowledgement (NAK) upon receipt of an incorrect command.

Serial Peripheral Interface

The platform uses the Serial Peripheral Interface (SPI) for communication between the Arduino Due and the SD card reader/ TFT display. SPI is a synchronous serial communication protocol that provides two-way master-slave type communication between a microcontroller and its peripherals. SPI facilities this communication using synchronization bits and preset data transfer speeds. An SPI interface communicates using at least four lines, with an additional line required for each additional peripheral device.

Inter-Integrated Circuit Communication

The platform uses Inter-Integrated Circuit Communication (I2C) to communicate between the Arduino Due and the RTC. I2C consists of serial data line (SDL) and a serial clock line (SCL). The data to be transferred is sent through the SDA wire and is synchronized with the clock signal from SCL. The standard formally defines signals connecting between a master device, in this case the Arduino Due, and a slave device, in this case the RTC.

Spectroradiometer Quality Indices

This section presents the Ocean Optics Flame-S spectroradiometer quality indices, as provided by the instrument manufacturer (Ocean Insight 2021).

Table S 2: The quality indices of the Ocean Insight Flame-S Spectroradiometer [2].

Quality Index	Description	
Wavelength uncertainty	<0.0001%	
Monochromatic pixel accuracy	at 210 nm:	16.2%
	at 250 nm:	10.8%
	at 300 nm:	10.4%
	at 350 nm:	11.4%
	at 400 nm:	8.0%
	at 450 nm:	5.8%
	at 500 nm:	4.6%
	at 550 nm:	3.8%
	at 600 nm:	3.4%
	at 650 nm:	3.0%
	at 700 nm:	2.8%
	at 750 nm:	2.8%
	at 800 nm:	2.8%
	at 850 nm:	2.8%
Spectral scatter	0.01%	
Slit scattering	<5%	
Signal to Noise (at saturation)	250:1	
Number of Pixels	2048	
Readout Noise	± 50 counts	
Pixel limit of detection	Min (200 nm):	6.72E-12 W
	Germicidal (254 nm):	1.59E-13 W
	Max (524 nm):	1.40E-14 W
Pixel saturation	Min (200 nm):	2.86E-02 W
	Germicidal (254 nm):	6.76E-04 W
	Max (524 nm):	5.98E-05 W
Sampling rate	1ms to 65s (platform range: 1ms to 5s)	

Optical Measurement Quality Indices

The primary quality indices that affect the measurement of UV radiation are the monochromatic pixel measurement uncertainty, spectral scatter, slit scattering, and signal to noise ratio [3]. Monochromatic pixel measurement uncertainty is the uncertainty inherent for a single pixel in the measurement of a monochromatic source. Spectral scatter and slit scatter cause out of band signal to arrive at each pixel. Spectral scatter refers to the diffuse radiation arriving at evenly distributed intensity at all pixels, proportional to the total radiation intensity present at the optical inlet. Slit scatter refers to the radiation scattered by the entrance slit of the spectroradiometer. Spectroradiometers with large slit scattering error display large full width half maximum

(FWHM) during measurement of monochromatic radiation, thus reducing the instrument's wavelength resolution.

Signal to noise ratio (SNR) is the signal intensity divided by the noise at a particular signal level. A spectroradiometer with a large signal to noise ratio can more accurately identify and measure a weak signal compared to one with a small signal to noise ratio [4].

The spectroradiometer contains eighteen pixels covered with an opaque material, called dark pixels, in addition to the 2048 optically active pixels. A manufacturer provided method to remove background noise generated by electrical and thermal fluctuations in the device subtracts the average signal generated by these dark pixels from the signal generated at each optically active pixel on the device. However, this method does not account for the noise introduced by spectral scatter (Ocean Insight 2015).

An uncorrectable source of noise originates in the detector's pre-amplifier during readout, aptly named readout noise. This spectroradiometer has a readout noise of ± 50 counts (Ocean Insight 2015).

The spectroradiometer has a variable limit of detection depending on the wavelength of the measured radiation. For an integration time at the device maximum integration time of 65 seconds, the device limit of detection ranges from 6.72E-12 W for 200 nm to 1.40E-14 W for radiation at 524 nm. The pixel saturation at the minimum integration time of 1ms is 2.86E-02 W at 200nm and 5.98E-05 W at 524 nm (Ocean Insight 2021).

Dynamic Integration Time Adjustment

The microcontroller adjusts the spectroradiometer integration time to target a pixel saturation level of 75%, adjusting based on the maximum number of signal counts collected for the previous collected measurement. The microcontroller first identifies the pixel with the maximum signal count, and uses it to generate a scaling multiplier with the following equation:

$$F = \frac{21363.6}{m} \quad (1$$

Where m is the maximum pixel signal count from the previously taken spectrum and F is a factor applied to the previous integration time as follows:

$$I_o = FI_i \quad (2$$

Where I_i is the previous integration time and I_o is the new integration time. If the new integration time exceeds 5 seconds, the microcontroller automatically sets the integration time to 5 seconds.

Processing Spectrum Data

Microcontroller Receipt of Data Words

Most Arduino libraries provide functions to handle bytes, not data words. To properly process data words sent by the spectroradiometer, the microcontroller applies a bit-wise operation to the data collected by the default Serial1.readBytes(buffer, size) function. This function collects a number of bytes equal to the 'size' parameter and stores them into the 'buffer' array. The bit-wise operation involves instantiating a data word using the bit-shift operator <<. Example C++ code illustrates this bit-wise operation.

```
byte byte_buf[2];
Serial1.readBytes(byte_buf, 2);
word data_word_A =  byte_buf[1] | (word) byte_buf[0] << 8;
```

The first line of this code allocates two bytes of the microcontroller's memory for the byte_buf array. The second line has the Serial1.readBytes function store the data word's MSB in byte_buf[1] and its LSB in byte_buf[0]. The third line initializes a data word variable named 'data_word_A' and uses the bit-shift operator to write the first 8-bits as the MSB and the second 8-bits as the LSB.

The microcontroller reads all 2056 data words sent by the spectroradiometer to flash memory. While the data exists in flash memory, the microcontroller performs several sequential processes to convert the data from raw signal counts into absolute irradiance in W/cm^2.

Linearity Correction

The microcontroller applies the manufacturer provided linearity correction calibration to each raw signal value in the spectrum. The value of each pixel undergoes the following function.

$$L_i = \frac{1}{F} C I_i$$

(3

Where L_i is the signal count of pixel *i* with the linearity correction applied, *C* is a constant equal to 65,535/28000 to scale the spectroradiometer raw signal into the manufacturer software scale of 0 to 65535, I_i is the uncorrected signal count of pixel *i*, and F is the factor computed from:

$$\begin{aligned} F = 0.884115 + & I_i * 1.00742 * 10^{-5} \\ & - {I_i}^2 * 5.39901 * 10^{-10} \\ & + {I_i}^3 * 2.78301 * 10^{-14} \\ & - {I_i}^4 * 1.10510 * 10^{-18} \\ & + {I_i}^5 * 2.59529 * 10^{-23} \\ & - {I_i}^6 * 3.05861 * 10^{-28} \\ & + {I_i}^7 * 1.34544 * 10^{-33} \end{aligned}$$

(4

Noise Correction

The manufacturer provided noise correction method averages the signal detected by each of 18 pixels, named dark pixels, that have an opaque coating that prevents the receipt of radiation signal. The average of these 18 pixels represents the average thermal and electrical noise inherent in a device measurement. The Arduino subtracts the average signal detected at these 18 pixels from the value measured at each of the other 2030 pixels present on the charge coupled diode.

Signal Thresholding

The microcontroller sets a pixel's corrected signal count value to zero if the value does not exceed a signal to a noise threshold. The threshold varies by wavelength band, and the noise value is the average computed during the previous noise correction process. For wavelengths under 315nm, the microcontroller requires the signal to be at least 66% larger than the noise. For wavelengths between 315 and 400nm, the signal needs to be at least 50% larger than the noise. For wavelengths between 400 and 871nm, the signal needs to be at least 10% larger than the noise.

Signal to Irradiance Conversion

The microcontroller uses manufacturer provided calibration factors along with the integration time, area of the spectroradiometer optical inlet, and the trapezoidal integration of the wavelength bounds to convert corrected signal counts into irradiance in µW/(cm^2*nm). To do so, the microcontroller uses the following function:

$$P_i = \frac{L_i * C_i}{0.119460cm^2 * t * \Delta x_i} \quad (5$$

Where P_i is the power in mW/(nm*cm^2), the constant is the area of the cosine corrector inlet in cm^2, L_i is the spectral scatter corrected pixel signal count, C_i is the calibration factor of pixel *i* in uJ/count, *t* is the integration time in seconds, and Δx_i is the wavelength bound in nm.

Supplemental Materials References

1. TIA *TIA/EIA-232 Standard Interface Between Data Terminal Equipment and Data Circuit-Terminating Equipment Employing Serial Binary Data Interchange*; 1997;
2. Ocean Insight *Flame-S Radiometric Calibration Report*; Orlando, 2021;
3. Nehir, M.; Frank, C.; Aßmann, S.; Achterberg, E.P. Improving Optical Measurements: Non-Linearity Compensation of Compact Charge-Coupled Device (CCD) Spectrometers. *Sensors* **2019**, *19*, doi:10.3390/s19122833.
4. Ocean Insight Glossary on Spectroscopy and Technical Terms Available online: https://www.oceaninsight.com/knowledge-hub/glossary/ (accessed on 8 August 2022).
5. Ocean Insight *Flame Miniature Spectrometer User Manual*; Orlando, 2015;
